# Zinc-finger protein ZNF165 is a novel cancer-testis antigen capable of eliciting antibody response in hepatocellular carcinoma patients

**DOI:** 10.1038/sj.bjc.6602138

**Published:** 2004-08-31

**Authors:** X-Y Dong, X-A Yang, Y-D Wang, W-F Chen

**Affiliations:** 1 Department of Immunology, School of Basic Medical Science, Peking University Health Science Center, 38 Xue Yuan Road, Beijing 100083, China

**Keywords:** zinc-finger protein, transcription factor, cancer-testis antigen, serum antibody, tumorigenesis

## Abstract

ZNF165 is a zinc-finger protein gene that was identified from human adult testis. Analysis of the origins of publicly available expressed sequence tags as presented in Unigene and SAGE databases revealed that ZNF165 mRNA was expressed in tumours of different tissues. RT–PCR, real-time PCR and Northern blotting analysis confirmed that ZNF165 mRNA was expressed in the hepatocellular carcinoma, gastric cancer, colon cancer and non-small-cell lung carcinoma. The nucleotide sequence of ZNF165 expressed in tumours is identical to that expressed in the testis. Humoral responses of hepatocellular carcinoma (HCC) patients against ZNF165 protein were determined by Western blotting using the recombinant ZNF165 protein. Antibodies against ZNF165 protein were detected in approximately 5% (four of 82) of the sera from HCC patients. These results suggest that ZNF165, a member of the ZNF family, is a novel CT antigen capable of eliciting humoral immune response and be involved in tumour biology.

Tumour development is a result of multiple genetic alterations. Tumour cell phenotype has six hallmarks: disregarding signals for growth arrest; failure to differentiate; capacity of sustained proliferation; evasion of apoptosis; ability of invasion; and enhanced angiogenesis (Bishop, 1987; Compagni and Christofori, 2000). A major challenge in tumour research is to illustrate how these phenotypic alterations result in tumorigenesis. Genes involved in promoting cellular proliferation are often altered in tumours; however, the mechanisms underlying the alternations are poorly understood. Many of these genes encode transcription factors that are the principal modulators of gene expression in the organism. Zinc-finger family of proteins is one of the most common families of transcription factors in eukaryotic cells and has more than 3000 members in the human genome. They are known to play a key role in regulating expression of genes important for cell growth, proliferation, differentiation and apoptosis (Cox and Goding, 1991; Papavassiliou, 1995; Ladomery and Dellaire, 2002). Owing to their multiple functions, some zinc-finger proteins are powerful regulators in the development of the tumour. For example, WT1 has been demonstrated to be responsible for the development of Wilm's tumour (Lee and Haber, 2001). The other well-characterised examples of zinc-finger proteins involved in tumorigenesis include those encoded by the genes of GLI, KS1, Evi9 and BCL11 subfamilies. However, the role of remainder members of the zinc-finger family in tumour development is not clear as yet (Dunaeva *et al*, 2003; Liu *et al*, 2003; Mitas *et al*, 2003). Therefore, investigation of additional zinc-finger proteins associated with tumorigenesis may provide insight into molecular mechanisms underlying tumorigenesis.

Hepatocellular carcinoma (HCC) is one of the most common and severe malignancies worldwide and its therapeutic results remain unsatisfactory. The identification of cancer-testis (CT) antigens recognised by cellular and/or humoral effectors of the immune system has provided new perspectives for HCC immunotherapy (Knuth *et al*, 2000). However, a crucial factor alleviating the efficacy of the immunotherapy is the immunoselection developed during the period of vaccine application and resulted in the progressive loss of tumour antigens (Giorgio *et al*, 2002). To improve the efficacy of tumour vaccines and decline immune escape, further identification of novel CT antigens is warranted for the development of applicable polyvalent tumour vaccines.

ZNF165, expressed in human adult testis, is a member of kruppel family of zinc-finger-containing transcription factors (Tirosvoutis *et al*, 1995). In this report, we have examined ZNF165 mRNA expression in tumours of different histological types using various approaches. We have found that ZNF165 mRNA is also expressed in the hepatocellular carcinoma, gastric cancer, colon cancer and non-small-cell lung carcinoma. Furthermore, antibodies against ZNF165 protein have been detected in the sera of some HCC patients. Thus, ZNF165 is a novel member of CT antigen family and may be both immunogenic and tumorigenic in HCC.

## MATERIALS AND METHODS

### Human tumour tissues

All samples of human HCC, gastric, colon and non-small-cell lung cancers and paired noncancerous tissue (5 cm away from tumour) were obtained during surgical resection from the 2nd Teaching Hospital, Peking University Health Science Center, China. Tissue samples were collected with patients' written consent and approved by Hospital Ethic Review Committee. The resected tissue samples were immediately cut into small pieces and snap frozen in liquid nitrogen until use. All tumour and paired noncancerous tissue samples were pathologically confirmed. The clinical stage of HCC patients was assessed according to the TNM classification of the International Union Against Cancer, and the histopathological grade of HCC differentiation was assessed according to the Edmondson Grading System. The patients from whom the resected HCC samples were obtained were HBV surface antigen positive but HCV antigen negative.

### RT–PCR

The mRNA expression pattern was determined using RT–PCR with a panel of commercially available cDNAs (Clontech, Palo Alto, CA, USA). The panel is composed of cDNAs generated from RNA isolated from 16 normal tissues: brain, heart, kidney, liver, lung, pancreas, placenta, skeletal muscle, colon, ovary, peripheral blood leucocyte, prostate, small intestine, spleen testis and thymus. RNA from human HCC, gastric, colon and non-small-cell lung cancers and paired noncancerous tissues was extracted and reverse transcribed with Advantage reverse transcriptase (Clontech, Palo Alto, CA, USA). Gene-specific PCR primers were used to amplify cDNA fragments of ZNF165. RT–PCR was performed with the parameters as follows: 30 cycles, 94°C, 15 s; 60°C, 30 s; and 72°C, 30 s, followed by 6 min at 72°C. Visualisation of target bands on a 1.2% agarose gel with ethidium bromide staining was performed to determine the expression of ZNF165 mRNA in the different types of tissues. The sequences of paired primers for the amplification of ZNF165 are: forward, ccc aga gag tgg aga gga ggc ag; and reverse, aac ctt cat cct ggg cag acg ag. To investigate if ZNF165 mRNA expressed in tumours is identical to that in the testis, a pair of primers spanning the open reading frame (ORF) of ZNF165 was designed and RT–PCR was performed using the template cDNAs from tumours and testis, respectively. The PCR amplification parameters were as follows: 30 cycles, 94°C, 15 s; 58°C, 30 s; and 72°C, 1.5 min, followed by 6 min at 72°C and the sequences of the primers: forward, caa gat ggc tac aga acc aaa gaa agc; and reverse, cat att gtg cca ttt cct cag cat tta c. The PCR products from tumours and testis were cloned and sequenced, respectively.

### Quantification of gene expression

Quantification of ZNF165 mRNA expression was performed by monitoring the increase in fluorescence by the binding of SYBR green to double-stranded DNA during real-time PCR. Their specific amplification primers were those described above. All reactions were carried out in triplicate with positive and negative controls using the standard program (the iCycler iQ Real-Time PCR Detection System, Bio-Rad Laboratories, California, USA). Melt-curve analysis was performed to determine the temperature at which data were collected. To collect data in real-time PCR, reaction mixtures were incubated at 50°C for 2 min and then 95°C for 10 min prior to 40 cycles of PCR (95°C for 15 s, 60°C for 30 s, 72°C for 30 s and 88°C for 10 s). Serial 1 : 10 dilutions of plasmid DNA containing ZNF165 cDNA (10^4^, 10^5^, 10^6^, 10^7^, 10^8^ and 10^9^ copies tube^−1^) were analysed and served as the standard curve. Copy number of ZNF165 cDNA was estimated by the standard curve. The specificity of PCR amplifications was confirmed by electrophoresis on a 1.2% agarose/EB gel. To determine the mRNA level of ZNF165, we used an mRNA EI, which indicates a relative level of mRNA expression standardised by G3PDH. The mRNA EI was calculated as following (in AU): mRNA EI=(copy number of ZNF165 mRNA/copy number of G3PDH mRNA) × 1000 AU.

### Northern blot analysis

Northern blotting was performed with mRNA samples extracted from HCC and paired noncancerous liver tissues. RNA integrity was examined by electrophoresis in formalin/4-morpholine propane sulphonic acid gels. In all, 10 *μ*g of mRNA per lane were first separated by electrophoresis in 1.2% agarose containing 3% formaldehyde and then blotted onto Hybond-C nylon membranes (Amersham). The membrane was crosslinked by ultraviolet radiation. The DIG-labelled probes were the cDNA sequence of ZNF165 (425 bp). After prehybridisation, the membrane was hybridised to specific DIG-labelled probes overnight at 42°C in hybridisation solution (50% formamide, 5 × SSC, 0.1% *N*-lauroylsarcosine, 0.02% SDS, 2% Blocking reagent and 100 *μ*g ml^−1^ sheared salmon sperm DNA). Filter was then sequentially washed at room temperature for 30 min in 0.1% SDS and 2 × SDS, and at 68°C for 30 min in 0.1% SDS and 0.1 × SDS. After stringent washes, the corresponding mRNA in Northern blotting was detected by chemiluminescence using CSPD ready-to-use reagent (Roche, Indianapolis, USA).

### Expression and purification of ZNF165 recombinant protein in *Escherichia coli*

Expression construct of ZNF165 was created by PCR amplification of the full-length cDNA with the 5′ and 3′ primers: gcg gga tcc atg gct aca gaa cca aag aaa gc and gcg gtc gac tgc cat ttc ctc agc att tac. The amplified products were digested with *Bam*HI and *Sal*I, and the fragment was inserted into the expression plasmid pET28a (+) followed by DNA sequencing. The recombinant plasmid pET28a/ZNF165 was transformed into *E. coli* BL-21 and the recombinant ZNF165 protein accounted for 15% of total protein. After the induction with 1 mM IPTG at 37°C for 6 h, the recombinant ZNF165 protein fused with 6 × His tag was purified by Ni^2+^ affinity chromatography.

### Western blotting

Purified recombinant ZNF165 protein was separated on 10% SDS–polyacrylamide gel electrophoresis and transferred onto nitrocellulose membranes. After blocking with the TNT solution containing 5% dry milk, the membranes were incubated with sera from normal individuals or HCC patients at a 1 : 500 dilution for 1.5 h, and then with goat anti-human IgG (H+L) alkaline phosphatase conjugate (Promega) for 1 h. Colour substrate NBT/BCIP (Promega) was added and incubated for 10 min for colour development.

## RESULTS

### Databases analysis

Unigene is an experimental processing system for automatically partitioning GenBank sequences into a nonredundant set of gene-oriented clusters. Each Unigene cluster contains sequences that represent a Unigene, as well as the related information such as the tissue types in which the gene is expressed. The Unigene incorporates hundreds of thousands of sequences of expressed sequence tag (EST) whose tissue distributions are labelled (Schuler, 1997). SAGE is an on-line technique that allows quantitation and comprehension of cellular gene expression profiling (Velculescu *et al*, 1995). Consequently, Unigene and SAGE can be used to analyse the spatial and temporal expression pattern of a given gene and predict the functional information of the gene. In the database of Unigene, there were 25 ESTs representing ZNF165. Although 14 of these ESTs were identified in the libraries of normal tissues such as the testis and placenta (10), pancreatic islet (3) and breast (1), the remainder 11 ESTs were derived from several types of tumours, such as uterus tumour, well-differentiated endometrial adenocarcinoma, leiomyosarcoma, germ cell tumour and carcinoid. In the SAGE database, one tag (AGGGAAAACC) representing ZNF165 was identified from the libraries of mammary gland carcinoma, prostate carcinoma and pancreas adenocarcinoma, respectively. These data suggest that ZNF165 mRNA has the expression characteristics typical of a CT antigen gene.

### Expression profile of ZNF165 mRNA

One of the criteria for identifying CT antigen genes is their specific expression in tumours, but not in normal tissues except the testis. The mRNA expression profile of ZNF165 was determined by RT–PCR. A series of tissues were examined, including normal tissues, HCC, gastric, colon and non-small-cell lung cancers and paired noncancerous tissues.

**Figure 1:**
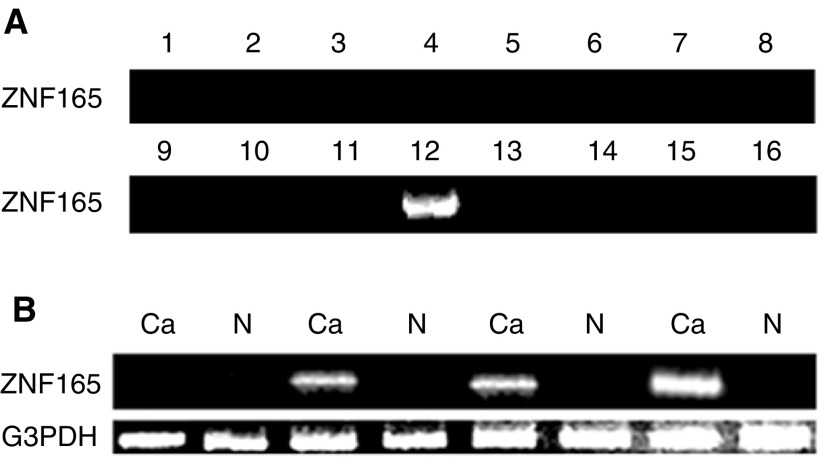
Expression patterns of ZNF165 mRNA in 16 normal tissues, HCC and paired noncancerous liver tissues. (**A**) Gel electrophoresis of RT–PCR products amplified by 30 cycles shows ZNF165 mRNA expression in 16 normal tissues. ZNF165 mRNA is only expressed in testis. 1–16 normal tissues: 1. brain; 2. heart; 3. kidney; 4. liver; 5. lung; 6. pancreas; 7. placenta; 8. skeletal muscle; 9. prostate; 10. ovary; 11. leucocyte; 12. testis; 13. small intestine; 14. spleen; 15. colon; 16. thymus. (**B**) Gel electrophoresis of RT–PCR products amplified by 30 cycles shows ZNF165 mRNA in the paired HCC and noncancerous liver tissue samples. RT–PCR for G3PDH was used to monitor the quality of the mRNA samples. Ca: HCC; N: paired noncancerous liver tissues.

**Figure 2:**
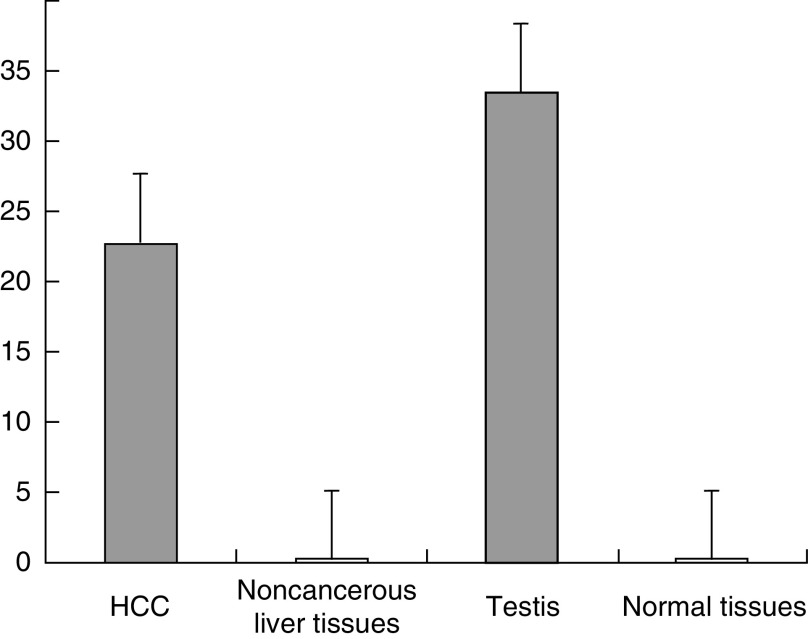
Quantification of ZNF165 mRNA expression in 22 HCC tissues, eight noncancerous liver tissues and normal tissues including the testis, placenta, lung, liver, pancreas, spleen, thymus and colon. Hepatocellular carcinoma: the average EI value is 22.81 in 22 HCC samples of ZNF165 mRNA positive; noncancerous liver tissues: the average EI value is 0.26 in eight noncancerous liver samples of ZNF165 mRNA positive; testis: the EI value is 33.56 in the testis; normal tissues: the average EI value is 0.24 in normal tissues including the placenta, lung, liver, pancreas, spleen, thymus and colon in which ZNF165 mRNA was expressed.

mRNA expression of ZNF165 was first examined by RT–PCR with the amplification by 30 cycles in 16 different normal tissues including the spleen, prostate, testis, ovary, small intestine, colon, peripheral blood, heart, lung, liver, whole brain, kidney, pancreas, placenta, thymus and skeletal muscle. ZNF165 mRNA expression was detected only in the testis (Figure 1A

). However, quantification of gene expression by real-time PCR with the amplification of 40 cycles revealed that ZNF165 mRNA could also be detected in the placenta, lung, liver, pancreas, spleen, thymus and colon, but not in other tissues. The average EI values of ZNF165 mRNA expression in the testis and other normal tissues including the placenta, lung, liver, pancreas, spleen, thymus and colon are 33.56 and 0.24, respectively. Therefore, the expression level of ZNF165 mRNA in these normal tissues was 140-fold lower than that in the testis (Figure 2

).

We then analysed ZNF165 mRNA expression in HCC and paired noncancerous liver tissues. The ZNF165 mRNA transcript was present in 22 of 42 HCC samples (52%) and eight of 42 paired noncancerous liver tissues. Representatives of such analyses are shown in Figure 1B. The average quantification of ZNF165 mRNA was 88-fold higher in HCC (the average EI value is 22.81 in 22 samples of ZNF165 mRNA positive) than in paired noncancerous liver tissues (the average EI value is 0.26 in eight samples of ZNF165 mRNA positive) (Figure 2). Of the 22 HCC samples of ZNF165 mRNA positive, there were 5, 6 and 11 samples belonging to well, moderate and poor differentiation status, respectively; there were 6, 8 and 8 samples belonging to clinic I–II, III and IV, respectively. Although the higher percentage of ZNF165 mRNA expression was observed in HCC patients with poor differentiation status, there was no correlation of the expression of ZNF165 mRNA with the clinic stage and tumour differentiation status of HCC patients (*P*>0.05).

To assess whether ZNF165 mRNA was expressed in tumours of different histological types, their mRNA levels in the gastric, colon and non-small-cell lung cancer tissues were tested. ZNF165 mRNA was expressed in six of 14 gastric cancer samples (43%), six of 14 colon cancer samples (43%) and in three of 14 non-small-cell lung cancer samples (21%). Thus, ZNF165 mRNA was expressed in tumours of various histological types and normal testis. Sequencing results of RT–PCR products showed that the nucleotide sequence of ZNF165 expressed in tumours was also identical to that expressed in the testis. Therefore, there was no mutation in the ORF sequence of ZNF165 expressed in tumours (data not shown).

### Seroreactivity of antibodies against ZNF165 protein in the sera of HCC patients

**Figure 3:**
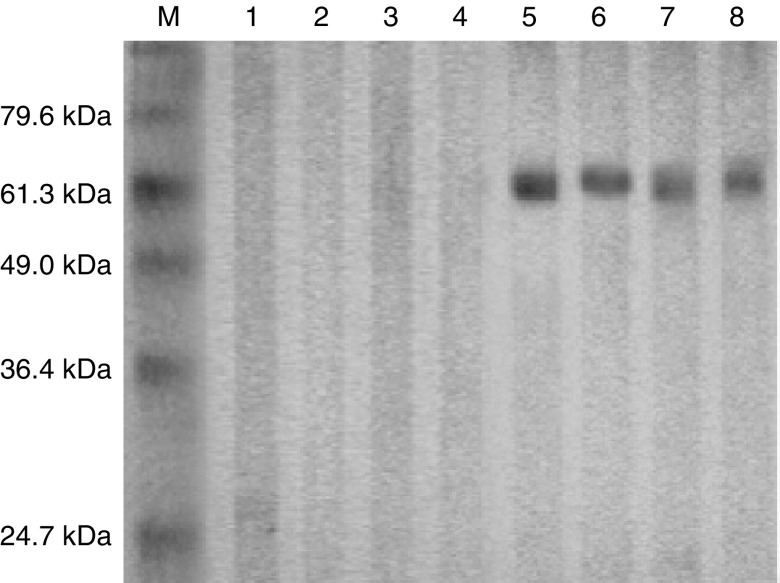
Western blotting of the positive sera against ZNF165 recombinant protein in HCC patients. In Western blotting, four sera from HCC patients were reactive to ZNF165 protein. In the sera from normal individuals, none was seroreactive to ZNF165 protein. The experiments were repeated twice and the same results were obtained. Lane M corresponds to protein marker; lanes 1–4 correspond to ZNF165 recombinant protein with four sera collected from normal individuals; lanes 5–8 correspond to the results of four positive sera reactive to ZNF165 recombinant protein.

A survey of the humoral immune response against ZNF165 antigen in HCC patients was performed by Western blotting. Of the 82 sera collected from HCC patients, four sera were reactive to ZNF165 protein (Figure 3

). Noticeably, although the expression of ZNF165 mRNA was not correlated with the clinic stage and tumour differentiation status of HCC patients, all the four seroreactive HCC patients were in the poorly differentiated status of clinic II (one of four), III (one of four) and IV (two of four) stage, respectively. As controls, in the 36 sera collected from normal individuals, none was reactive to ZNF165 protein (Figure 3). Moreover, sera collected from HCC, whose resected tumour tissue samples did not express ZNF165 mRNA, were serologically negative to ZNF165 protein.

## DISCUSSION

In this study, we have demonstrated that ZNF165, a gene identified from adult testis, is a novel CT antigen gene. The mRNA expression of ZNF165 has been found in human HCC, gastric, colon and non-small-cell lung cancers.

Examination of ZNF165 mRNA expression pattern by RT–PCR with 30 cycles revealed that ZNF165 was not detected in normal tissues except the testis. Under real-time PCR, the ZNF165 mRNA was not only expressed in tumours and normal testis but also could be detected in paired noncancerous tissues and some normal tissues including the placenta, lung, liver, pancreas, spleen, thymus and colon. Considering that some CT antigen genes like NY-ESO-1 and MAGE-A3 are reported to be detected in normal tissues (Scanlan *et al*, 2002) and we have also confirmed that NY-ESO-1 mRNA was detected in normal liver tissues by real-time PCR, our data support that ZNF165 is a novel CT antigen. Like most of the other CT antigens capable of inducing the humoral immune response and the cellular immune response in a minor proportion and a considerable proportion of cancer patients, respectively (Jager *et al*, 2001; Kirkin *et al*, 2002), the ZNF165 antigen spontaneously elicited antibody response detected in approximately 5% of HCC patients. There was no serological reactivity detected in the sera of healthy blood donors. Since the majority of ZNF165 antibodies detected are of the IgG class, the activation of CD4^+^ T-cell response to ZNF165 antigen could be present (Kennedy *et al*, 2003). Further experiments are ongoing to test if the ZNF protein could provoke cytotoxic T-cell response in cancer patients.

The results of Northern blotting further confirmed that ZNF165 mRNA was expressed in tumours (data not shown). ZNF165 mRNA detected in tumours is 2.2 kb, consistent with that found in the testis (Tirosvoutis *et al*, 1995). The ORF of ZNF165 cDNA encodes a predicted protein of 485 amino-acid residues. This protein has an N-terminal acidic domain containing 17% aspartic and glutamic acid residues and a C-terminal domain with five conserved C2H2-type zinc-finger motifs. ZNF165 is predicted to be a nuclear protein with an isoelectric point of 7.0. There were three N-myristoylation sites, one N-glycosylation site and several kinase phosphorylation sites. Thus, ZNF165 may undergo post-translational modifications. By comparing the cDNAs to the human genome, ZNF165 gene was located to chromosome 6p21.3, where a cluster of zinc-finger family members is located, such as ZNF184 and ZNF345. Members of this DNA-binding protein family act as transcriptional regulators.

Apart from a few zinc-finger proteins, such as WT1, BCL11A, Evi9, EWS, TLS, GLI, and CTCF, the function of the majority of zinc-finger proteins in tumour occurrence and development is unknown (Lee and Haber, 2001; Klenova *et al*, 2002; Liu *et al*, 2003; Dunaeva *et al*, 2003; Shing *et al*, 2003; Zwerner *et al*, 2003). As a zinc-finger protein containing an N-terminal acidic domain and a C-terminal domain with five conserved C2H2-type zinc-finger motifs, the ZNF165 may function in the transcriptional regulation. Transcription factors are the principal modulators of gene expression in the normal tissue development as well as in tumorigenesis once their function or expression pattern is altered. It remains to be determined whether the expression of ZNF165 is involved in tumorigenesis and progression. Answers to these questions will help to further understand tumour formation.
